# Bacteraemic urinary tract infections in a tertiary hospital in Japan: the epidemiology of community-acquired infections and the role of non-carbapenem therapy

**DOI:** 10.1186/s13104-017-2680-z

**Published:** 2017-07-27

**Authors:** Momoko Mawatari, Kayoko Hayakawa, Yoshihiro Fujiya, Kei Yamamoto, Satoshi Kutsuna, Nozomi Takeshita, Norio Ohmagari

**Affiliations:** 1 National Center for Global Health and Medicine, Disease Control and Prevention Center, Toyama 1-21-1, Shinjuku, Tokyo 162-8655 Japan; 2 Gunma University Hospital, Infection Control and Prevention Center, Showa 3-39-15, Maebashi, Gunma 371-8511 Japan

**Keywords:** ESBL-producing *Enterobacteriacea*e, Urinary tract infection, Non-carbapenem β-lactam, Community

## Abstract

**Objectives:**

This study aimed to describe the epidemiology of bacteraemic urinary tract infections (UTIs), especially those that were community-acquired (i.e., with no discernible healthcare-associated exposure) and caused by extended-spectrum beta-lactamase-producing *Enterobacteriaceae* (ESBLPE). We also evaluated and compared empirical antimicrobial treatments [carbapenem (CBP) vs. non-carbapenem beta-lactam (non-CBPBL)] for bacteraemic UTIs. Finally, we reviewed the published literature on the effectiveness of non-CBP compared to CBP treatments for UTIs caused by extended-spectrum beta-lactamase-producing organisms.

**Results:**

A total of 339 bacteraemic UTI episodes were identified; 32 (9.4%) were caused by ESBLPE. In bacteraemic UTI episodes, ESBLPE accounted for 8.3% of hospital-acquired cases, 10.0% of community-acquired cases, and 8.2% of non-healthcare-associated cases. As effective empirical therapy for ESBLPE, 12 patients received CBP and 7 patients received non-CBPBL treatments [piperacillin/tazobactam (PT) or cefmetazole (CMZ)]. Age, sex, Pitt bacteraemia score, immunosuppressive status, and causative bacterial species were similar between groups; neither group experienced mortality within 14 days. The number of days to defervescence was similar between groups. No difference was noted in the rates of microbiological cure (58% vs. 57%, P = 1.0). Five of seven patients in the non-CBPBL group did not receive CBP during the treatment period, even as definitive therapy, but all experienced clinical cure.

## Introduction

The increase in extended-spectrum beta-lactamase-producing *Enterobacteriaceae* (ESBLPE) is a considerable public health issue for various clinical fields. The Japan Nosocomial Infections Surveillance (JANIS) reported that third-generation cephalosporin-resistant *Escherichia coli* was detected in 92% of hospitals in 2014 [1]. In addition, community-acquired (CA) infections caused by ESBLPE have recently been increasing in Japan [2]; however, the epidemiology and burden of such infections, and their appropriate clinical management, remain unclear.

Several studies have shown that the optimal treatment for ESBLPE infections could differ depending on each patient’s background factors [3–5]. Carbapenem (CBP) overuse might cause an increase in multi-drug-resistant bacterial infections; therefore, effective alternatives to CBP should be determined for use in specific clinical scenarios.

The present study determined the epidemiology of bacteraemic urinary tract infections (UTIs), focusing on CA (i.e., with no discernible healthcare-associated exposure) infections caused by ESBLPE. We also evaluated and compared empirical antimicrobial treatments (CBP vs. non-carbapenem beta-lactam [non-CBPBL]) for bacteraemic UTIs and performed a review of the published literature on this issue.

## Main text

### Methods

#### Study setting and design

We conducted a retrospective observational study at the National Center for Global Health and Medicine (NCGM), a tertiary hospital with 780 beds. Patients were included if they were >15 years of age and had been diagnosed with bacteraemia due to UTIs between April 2012 and March 2015. If the same patient had multiple episodes of bacteraemia due to a UTI, only episodes occurring at least 30 days after the end of treatment for the previous UTI were counted as different episodes. Patients’ medical charts were reviewed by infectious diseases physicians to collect information on demographics, underlying diseases, clinical courses (including treatment given), and laboratory data (including microbiology). The BACTEC 9240 and BACTEC FX blood culture systems (Becton–Dickinson, MD, USA) were used to process blood specimens. Isolate identification and susceptibility testing were performed using the MicroScan Walkaway 96 SI system (Siemens Healthcare Diagnostics, Tokyo, Japan), and the minimum inhibitory concentrations were interpreted using the Clinical and Laboratory Standards Institute (CLSI) criteria [6]. We also reviewed published studies that compared outcomes for CBP and non-CBP treatments for UTIs caused by ESBLPE. This study was approved by the NCGM’s institutional review board before the study’s initiation (NCGM-G-001790-00).

#### Definitions and study end points

UTIs included any infection of the urinary system, including pyelonephritis, renal abscess, cystitis, prostatitis, and urinary device-related infections.

Hospital-acquired (HA) events were defined as infections occurring on or after the 4th day of hospitalization. CA events were defined as infections occurring within 3 days after admission. Among CA events, non-healthcare-associated (NHCA) events were further categorized and defined as follows: CA events for patients that were not hospitalized (≥2 days) in an acute care hospital within 90 days, who were not living in a nursing home, and who did not require home-visit nursing, intravenous therapy, wound care, or haemodialysis within 30 days. Non-CBPBL treatment consisted of either piperacillin–tazobactam (PT) or cefmetazole (CMZ).

Empirical therapy was defined as antibiotic therapy administered at the time that the blood culture was obtained until microbiological susceptibility data became available. Effective empirical therapy was defined as empirical therapy to which the causative pathogens were susceptible, based on the CLSI criteria [6]. Definitive therapy was defined as antibiotic therapy administered after microbiological susceptibility data became available.

Patients were defined as clinically cured when they became afebrile and when a physician judged the infection to be healed. Microbiological cure was defined when blood or urine cultures became negative after the antibiotics were begun and when there was no recurrence of infection or colonization of ESBLPE.

Bacteraemia severity was assessed at the time of the first positive blood culture using the Pitt bacteraemia score, a scoring system based on mental status, vital signs, mechanical ventilation, and recent cardiac arrest [7].

#### Statistical analysis

Mann–Whitney U tests were used to compare continuous variables, and χ^2^ or Fisher’s exact tests were used to compare categorical variables. Odds ratios and 95% confidence intervals were calculated by comparing the categorical variables of characteristics of CBP and non-CBPBL treatments. All *P* values were two-sided, and *P* < 0.05 were considered statistically significant. All statistical analyses were performed using EZR (Saitama Medical Center, Jichi Medical University, Saitama, Japan) [8].

### Results

**Fig. 1:**
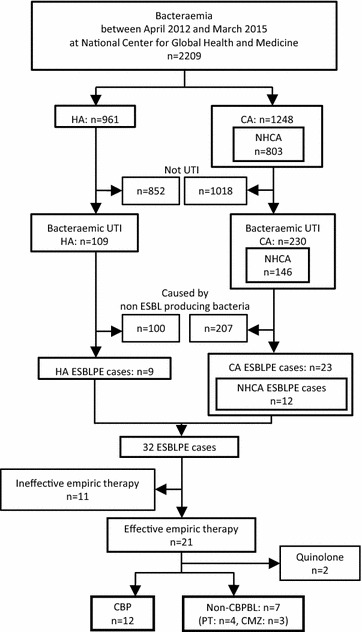
Schematic of patient enrolment. *HA* hospital-acquired, *CA* community-acquired, *NHCA* non-healthcare associated, *UTI* urinary tract infection, *ESBL* extended-spectrum beta-lactamase, *ESBLPE* extended-spectrum beta-lactamase-producing *Enterobacteriaceae*, *CBP* carbapenem, *non-CBPBL* non-carbapenem beta-lactam, *PT* piperacillin–tazobactam, *CMZ* cefmetazole

A total of 339 bacteraemic UTI episodes, caused by 372 pathogens, were identified (Table 1; Fig. 1). Of the bacteraemic UTI episodes, 32 (9.4%) were caused by ESBLPE (*E. coli*, 27; *Klebsiella* spp. 5). ESBLPE accounted for 8.3% (9/109) of HA cases, 10.0% (23/230) of CA cases, and 8.2% (12/146) of NHCA cases. There was no significant difference observed in the proportion of ESBLPE among these groups (P = 0.79). Among ESBLPE causing bacteraemia in patients with UTIs, ESBL-*E. coli* was the dominant pathogen (n = 27, 84%), followed by *K. pneumoniae* (n = 4, 12.5%), and *K. oxytoca* (n = 1, 3.1%).

**Table 1:** Pathogens isolated from patients with bacteraemia due to urinary tract infections

	All		HA		CA		NHCA	
	N	%	N	%	N	%	N	%
*Escherichia coli* (non-ESBL)	164	44.1	44	38.3	120	46.7	45	64.3
*Escherichia coli* (ESBL)	27	7.3	7	6.1	20	7.8	6	8.6
*Klebsiella pneumoniae* (non-ESBL)	33	8.9	13	11.3	20	7.8	4	5.7
*Klebsiella pneumoniae* (ESBL)	4	1.1	2	1.7	2	0.8	0	0.0
*Klebsiella oxytoca* (non-ESBL)	9	2.4	4	3.5	5	1.9	1	1.4
*Klebsiella oxytoca* (ESBL)	1	0.3	0	0.0	1	0.4	1	1.4
Other *Enterobacteriaceae*	38	10.2	15	13.0	34	13.2	6	8.6
Non-fermenting gram-negative rods	22	5.9	11	9.6	6	2.3	0	0.0
Gram-positive cocci	58	15.6	18	15.7	39	15.2	7	10.0
Others	16	4.3	1	0.9	10	3.9	0	0.0
Total	372		115		257		70	
Proportion of ESBLPE	9.4%		8.3%		10.0%		8.2%	

HA, hospital-acquired; CA, community-acquired; NHCA, non-healthcare associated; ESBLPE, extended-spectrum beta-lactamase-producing *Enterobacteriaceae*

Twenty-one patients received effective empirical therapy for ESBLPE (Fig. 1): 12 (57%) patients received CBP; 4 (19%), PT; 3 (14%), CMZ; and 2 (10%), quinolones, respectively. The characteristics of CBP and non-CBPBL treatments were compared (Table 2). Age, sex, Pitt bacteraemia score, and causative bacterial species were similar between groups. The duration of the previous hospital stay tended to be longer in the CBP group than in the non-CBPBL group (P = 0.06). The rate of patients with any immunosuppressive condition was not different between groups (P = 0.62); however, there were more patients with malignancies in the non-CBPBL group (P = 0.04). Nine (75%) in the CBP group and 2 (29%) in the non-CBPBL group were administered CBP as definitive therapy after the pathogens and their sensitivities to antibiotics were determined.

**Table 2:** Characteristics of patients with bacteraemia due to urinary tract infections caused by ESBL-producing *Enterobacteriaceae*

	CBP, N = 12	non-CBPBL, N = 7	OR* (95% CI)	P*
Age, mean [±SD]	76.7 [±7.2]	72.9 [±18.1]	NA	0.9
Male sex	2 (16.7)	3 (42.9)	0.3 (0.02–3.5)	0.31
Nursing home resident	6 (50)	1 (14.3)	5.5 (0.4–320.4)	0.17
Hospital-acquired	5 (41.7)	0 (0)	NA	0.11
Community-acquired	7 (58.3)	7 (100)	NA	0.11
Non-healthcare-associated	2 (16.7)	3 (42.8)	0.3 (0.02–3.5)	0.31
Antibiotics use within the previous 3 months	4 (40)^a^	4 (57)	1.1 (0.1–11.6)	1.0
Underlying diseases related to the urinary tract	2 (16.7)	3 (42.9)	0.3 (0.02–3.5)	0.31
History of UTI	8 (66.7)	2 (28.6)	4.6 (0.5–69.2)	0.17
Urinary catheter use	4 (33.3)	1 (14.3)	2.8 (0.2–171.8)	0.6
Previous hospital days, mean [SD]	90 [±156.6]	0	NA	0.06
Any immunosuppressive condition^b^	9 (75)	4 (57)	2.2 (0.2–24.7)	0.62
Diabetes mellitus	3 (25)	1 (14.3)	1.9 (0.1–122.1)	1.0
Malignancy	1 (8.3)	4 (57.1)	0.1 (0.001–1.2)	0.04
Pitt bacteraemia score, median [IQR]	4 [2–5]	3 [2–4]	NA	0.57
Causative bacteria species				
*Escherichia coli*	11 (91.7)	6 (85.7)	1.8 (0.02–156.6)	1.0
*Klebsiella pneumoniae*	1 (8.3)	1 (14.3)	0.6 (0.006–49.9)	1.0
Use of CBP as definitive therapy	9 (75)	2 (29)	21.1 (1.4–1395.7)	0.01

Data were compared between groups treated with empirical treatments (carbapenem vs. non-carbapenem beta-lactam). Values are number (%) unless otherwise indicated

ESBL, extended-spectrum beta-lactamase; CBP, carbapenem; non-CBPBL, non-carbapenem beta-lactam; OR, odds ratio; CI, confidence interval; SD, standard deviation; UTI, urinary tract infection; IQR, interquartile range; NA, not available

* The sample size was not large enough to conduct accurate statistical analysis; thus, caution is necessary when interpreting the results

^a^Information was available for only 10 patients

^b^Any of the following: use of immunosuppressive agents, presence of diabetes mellitus, malignancy, or chronic renal failure

No patient from either group died within 14 days after bacteraemia (Table 3). The number of days to defervescence were similar between groups (CBP: median = 1.5 [interquartile range 1–4]; non-CBPBL: 2 [1, 2]). The length of the hospital stay after bacteraemia tended to be longer in the CBP group (24 days [15–132] vs. 13 days [12–16], P = 0.08). No statistically significant difference was noted in the rates of clinical cure or microbiological cure (7/12 [58%] vs. 4/7 [57%], P = 1.0). Five of seven patients in the non-CBPBL group did not receive CBP during the treatment period, but all experienced clinical cure.

**Table 3:** Outcomes of patients with bacteraemia due to urinary tract infections caused by ESBL-producing *Enterobacteriaceae*

	CBP, N = 12	non-CBPBL, N = 7	P value*
14-day mortality	0%	0%	NA
Days to defervescence, median [IQR]	1.5 [1–4]	2 [1, 2]	0.74
Length of hospitalization after bacteraemia, median days [IQR]	24 [15–132]	13 [12–16]	0.08
Clinical cure	12 (100%)	6 (85.7%)	0.37
Microbiological cure	7 (58%)	4 (57%)	1.0

Data were compared between groups treated with empirical treatments (carbapenem vs. non-carbapenem beta-lactam)

ESBL, extended-spectrum beta-lactamase; CBP, carbapenem; non-CBPBL, non-carbapenem beta-lactam; IQR, interquartile range; NA, not available

* The sample size was not large enough to conduct accurate statistical analysis; thus, caution is necessary when interpreting the results

The studies we reviewed are shown in Table 4. Studies #1 [9], #2 [10], #7 [5], and #10 [11] showed that CBP was superior for treating ESBLPE bacteraemia, but studies #1 and #2 included non-CBP agents, including some antibiotics other than beta-lactam/beta-lactamase inhibitors (BLBLIs) and cephamycin. Studies #7 and #10 had fewer than 25% of UTIs among the total infections in the group. CBP was not significantly superior to non-CBP agents in the studies that included 40% or more of UTIs among the total infections (studies #4–6 [4, 12, 13], #8 [14], #9 [15], #11 [16]).

**Table 4:** Studies on the effectiveness of non-carbapenem versus carbapenem treatment for UTIs due to ESBL-producing organisms

No	Author, year	Country	Enrolment criteria	Source of infection	Study design	Non-CBP agents	Compared therapy	Pathogen	Outcome	Result
#1	Paterson, 2004	Seven countries^a^	BSI	UTI 14%	Prospective	Any	DT	KP	28-day mortality	CBP was superior
#2	Lee, 2010	Taiwan	BSI	UTI 7.4%	Retrospective	BL	DT	*Enterobacter cloacae*	Mortality	CBP was superior
#3	Rodriguez-Bano, 2012	Spain	BSI	UTI and biliary tract infection 70%	Post-hoc analysis	BLBLI	ET, DT	EC	Mortality	NS
#4	Doi, 2013	Japan	Bacteriuria	Only UTI	Retrospective	CMZ	Through ET and DT	Any	Clinical and microbiological cure	NS
#5	Kelvin, 2013	China	BSI	UTI 44%	Retrospective	Any	ET, DT	EC	30-day mortality	NS
#6	Park, 2014	Korea	Pyelonephritis	Only UTI	Retrospective	Any	Through ET and DT	EC	Clinical failure	NS
#7	Tamma, 2015	US	BSI	UTI 19%	Retrospective	PT	ET	Any	14-day mortality	CBP was superior
#8	Harris, 2015	Singapore	BSI	UTI 47%	Retrospective	BLBLI	DT	EC and KP	30-day mortality	NS
#9	Matsumura, 2015	Japan	BSI	UTI 45%	Retrospective	CMZ, FMOX	ET, DT	EC	30-day mortality, clinical response	NS
#10	Lee, 2015	Taiwan	BSI	UTI 23%	Retrospective	FMOX	DT	EC and KP	30-day mortality	CBP was superior
#11	Tsai, 2015	Taiwan	BSI	UTI 51%	Retrospective	PT	DT	*Proteus mirabilis*	30-day mortality	NS

UTIs, urinary tract infections; ESBL, extended-spectrum beta-lactamase; CBP, carbapenem; BSI, blood stream infection; DT, definitive therapy; KP, *Klebsiella pneumoniae*; BL, β-lactam; BLBLI, β-lactam with β-lactamase inhibitor; ET, empiric therapy; EC, *Escherichia coli*; NS, non-significant difference; CMZ, cefmetazole; FMOX, flomoxef; PT, piperacillin/tazobactam

^a^South Africa, Taiwan, Australia, Argentina, US, Belgium, and Turkey

### Discussion

The reported rates of CA ESBLPE are increasing worldwide [17]. Chong et al. reported that the carriage rate of ESBLPE among outpatients in a single Japanese hospital was 1.0% in 2003 and 13.7% in 2011 [2]. Over 90% of their samples were urine samples. In our study, ESBLPEs were isolated from bacteraemic UTI patients in 12 (8.2%) out of 146 NHCA bacteraemia cases and 9 (8.3%) out of 109 HA bacteraemia cases. The lower rate of ESBLPE in this study than in Chong’s report is possibly due to differences in the denominators (outpatients vs. bacteraemic patients), isolation sites (urine vs. blood), and study locations.

In the JANIS 2014 report, the rate of third-generation cephalosporin-resistant bacteria was 14.8% for *E. coli* and 5.6% for *K. pneumoniae* [1]. Our study showed that the rate of ESBL-producing *E. coli* was 14.1% for total cases of isolated *E. coli*, and of ESBL-producing *K. pneumoniae* was 10.8% of the total isolated cases of *K. pneumoniae*. Although the rate of ESBL-producing *K. pneumoniae* in our hospital was higher than that in the JANIS reports, no NHCA bacteraemia was caused by ESBL-producing *K. pneumoniae* in this study. The JANIS network consists of 883 hospitals, including approximately 70% of smaller hospitals with less than 500 beds. The rate of ESBL-producing *K. pneumoniae* might have been higher in our study due to the location of the hospital (in the centre of urban Tokyo) and the function of the hospital, which serves as a tertiary referral centre for severe patients.

Our report showed similar rates of ESBLPE between HA cases and NHCA cases. This finding suggests the spread of ESBLPE, especially ESBL-*E. coli*, to the Japanese community, and even to the people who had no healthcare exposure. The study results showed that about 10% of bacteraemic UTIs were caused by ESBLPE in both outpatient and inpatient cases, for which we must carefully consider the appropriate empirical therapy. CBP is a reliable treatment option for bacteraemia due to ESBLPE; however, the increase in CBP-resistant organisms worldwide, including in Japan, is a serious concern, for which the use of CBP is a known risk factor [18].

Previous studies (Table 4) that compared the effectiveness of CBP and non-CBP treatments varied in the definitions they used, the proportion of UTIs, and the types of therapies included (i.e., empirical and/or definitive). Study #4 included only UTIs and suggested that cefmetazole might be an alternative to CBP. Study #4 recruited patients diagnosed with pyelonephritis by clinicians based on bacteriuria and pyuria; therefore, the definition of infections might be ambiguous. Our study used stricter definitions, such as including only bacteraemic UTIs, and showed that the rates of clinical cure and microbiologic cure were not different between the CBP and non-CBPBL groups. Moreover, a comparison of characteristics of patients in the CBP and non-CBP groups in our study did not suggest that patients in the CBP group were sicker or had more comorbidities. Studies #8, #9, and #11 included bloodstream infection cases and compared CBP and non-CBP treatments, and over 40% of cases were UTIs. Studies #8 and #11 compared CBP and BLBLI given as definitive therapy. The proportion of cases receiving inappropriate agents as empirical therapies in the CBP and BLBLI groups was similar to that in #8 (35 and 37%, respectively). In Study #11, that proportion was unclear. To reduce mortality, rapid initiation of effective antibiotic coverage for severe sepsis and septic shock has been recommended [19], and thus, empirical therapy is considered to be a key factor for improving outcomes in blood stream infections. We, therefore, conducted a comparison of CBP and non-CBPBL treatment as an empirical therapy for bacteraemic UTIs and excluded cases of patients who received inappropriate empirical therapy. All cases in our study received appropriate definitive therapy. As definitive therapies were chosen by physicians according to susceptibility reports, various agents were used in our cohort (such as BLBLI, CBP, CMZ, fluoroquinolone, and sulfamethoxazole/trimetprim). Five patients reached clinical cure without receiving CBP at all.

According to our findings, which are in line with those of previously published studies, non-CBPBL treatment, such as PT and CMZ, might be a reasonable alternative to CBP in patients with bacteraemia due to a UTI. Under the current situation of increasing ESBLPE in the community in worldwide, non-CBPBL treatment should be considered as an option for empirical therapy for patients with UTIs.

This was a retrospective observational study, and most of the isolates included in this study were not available for further microbiological/molecular analysis. Although 8 out of 27 ESBL-*E. coli* isolates included in this study were found to be positive for different groups of CTX-M (4 isolates were positive for CTX-M group 9, 1 isolate was positive for CTX-M group 1 [other than CTX-M-15] [20], and 3 isolates were positive for CTX-M-15), it is possible that a closely related clonal strain might have caused some portion of the ESBLPE included in this study.

In conclusion, we found that the rates of ESBLPE in patients with bacteraemic UTIs were similar among HA, CA, and NHCA cases. CMZ and PT seem to be safe and effective alternatives to CBP as empirical therapies for bacteraemic UTIs.

## Limitations

The sample size was not large enough to conduct accurate statistical analysis; thus, caution is necessary when interpreting the results.

## Abbreviations

BLBLI: beta-lactam/beta-lactamase inhibitor
CA: community-acquired
CBP: carbapenem
CLSI: Clinical and Laboratory Standards Institute
CMZ: cefmetazole
ESBL: extended-spectrum beta-lactamase
ESBLPE: extended-spectrum beta-lactamase-producing *Enterobacteriaceae*
HA: hospital-acquired
JANIS: Japan Nosocomial Infections Surveillance
Non-CBPBL: non-carbapenem beta-lactam
UTI: urinary tract infection
